# Process evaluation of the digital Health4Life intervention among a sample of disadvantaged adolescents and teachers

**DOI:** 10.1093/heapro/daae170

**Published:** 2024-11-29

**Authors:** Lyra Egan, Lauren A Gardner, Nicola C Newton, Katrina E Champion

**Affiliations:** The Matilda Centre for Research in Mental Health and Substance Use, Level 6, Jane Foss Russell Building, The University of Sydney, Sydney, NSW 2006, Australia; The Matilda Centre for Research in Mental Health and Substance Use, Level 6, Jane Foss Russell Building, The University of Sydney, Sydney, NSW 2006, Australia; The Matilda Centre for Research in Mental Health and Substance Use, Level 6, Jane Foss Russell Building, The University of Sydney, Sydney, NSW 2006, Australia; The Matilda Centre for Research in Mental Health and Substance Use, Level 6, Jane Foss Russell Building, The University of Sydney, Sydney, NSW 2006, Australia

**Keywords:** process evaluation, sociodemographic disadvantage, adolescent health, lifestyle behaviours, prevention, eHealth

## Abstract

Disadvantaged adolescents, including those from lower socioeconomic status (SES) or geographically remote backgrounds, engage in higher rates of risk behaviours, including poor diet, alcohol and tobacco use. While digital interventions targeting lifestyle risk behaviours show potential, few studies have focused on their implementation and relevance for this population. This study conducted a process evaluation of ‘Health4Life’, a universal school-based digital program targeting multiple behaviours, among a sample of disadvantaged adolescents. Participants were from six schools classified as low SES (Index of Community Socio-educational Advantage percentile score ≤ 25%), and/or regional using the Australian Statistical Geography Standard. Self-reported student (*n* = 214) and teacher evaluations (*n* = 16) assessed Health4Life’s acceptability, with qualitative questions capturing areas for improvement. Teacher-reported implementation data (*n* = 16) measured intervention fidelity and feasibility. Quantitative data were analysed using descriptive statistics and open-ended responses were thematically analysed. Compared to the entire sample, this subset of students evaluated Health4Life less favourably (66% versus 75%), with fewer enjoying the stories (63% versus 75%) and planning to use the skills and information (60% versus 70%). Teacher evaluations were mostly positive and aligned closely with the entire sample. Fidelity data also indicated comparable levels of student engagement (~92% versus ~85%). Key themes for refinement included improving content relevance and technical execution to better resonate with disadvantaged adolescents. While teacher evaluations suggest Health4Life is a valuable program in low SES or regional contexts, students’ lower ratings indicate refinements are needed. Identified areas for improvement will guide co-designing the program’s adaptation to improve effectiveness and relevance for disadvantaged adolescents.

Trial registration: The Health4Life trial is registered with the Australian New Zealand Clinical Trials Registry (ACTRN12619000431123).

**Contribution to Health Promotion**

- While digital interventions show potential for targeting lifestyle risk behaviours among disadvantaged adolescents, few studies have focused on their implementation and relevance for this population.
- To address this gap, this study conducted a process evaluation of *Health4Life*, a universal multiple health behaviour change intervention, among a sample of disadvantaged adolescents and their teachers.
- The findings highlight the need to adapt and optimize interventions through place-based co-design approaches to ensure effectiveness and relevance to disadvantaged populations.
- This study provides critical insights into designing and delivering interventions for different subgroups, including low SES and regional adolescents.

## INTRODUCTION

Research increasingly demonstrates that social determinants, including the contexts in which a person is born, raised and lives, as well as their occupation and access to health resources, are linked to health outcomes (Commission on Social Determinants of Health, 2008; Viner *et al*., 2012; Spencer *et al*., 2019; Flavel *et al*., 2024) including chronic diseases such as cancer, substance use disorders and mental ill-health (Marmot and Bell, 2019; Massouh *et al*., 2023). Disadvantaged adolescents, including those of low socioeconomic status (SES) or living in geographically remote areas, are particularly vulnerable to experiencing greater chronic disease burden and tend to report higher rates of modifiable lifestyle risk behaviours, including poor diet, and alcohol and tobacco use, than their more advantaged peers (Australian Institute of Health and Welfare 2020; Gautam *et al*., 2023). These behaviours are among the highest contributors to chronic disease morbidity and mortality in adulthood and often manifest during adolescence (Akseer *et al*., 2020). Thus, preventing disadvantaged adolescents’ engagement in these modifiable lifestyle risk behaviours is a public health priority and is critical for safeguarding their health and reducing healthcare burden and health inequities.

To achieve this, surmounting social, structural and economical barriers to access and participation in preventive interventions for disadvantaged adolescents is critical (Alliott *et al*., 2022). School-based digital interventions, delivered through various platforms including computers, internet, mobiles or telephones, leverage adolescents frequent use of technology to reach them in a space they are already engaged with (Australian Bureau of Statistics, 2016–17; Patton *et al*., 2016; Vogels *et al*., 2022). Compared to traditional face-to-face interventions, digital interventions overcome socioeconomic and geographic barriers to access by offering accessible and convenient access to evidence-based content, with increased cost-effectiveness and potential for enhanced engagement and fidelity (Newton *et al*., 2017). Despite evidence supporting their effectiveness among the general population (Champion *et al*., 2019; Hutton *et al*., 2020; Newton *et al*., 2022), it is essential to tailor digital interventions to the needs and skills of disadvantaged adolescents to promote effective engagement (Yardley *et al*., 2016). This is crucial for facilitating disadvantaged adolescents’ participation, particularly as they already face unique challenges to health including stigma (Robards *et al*., 2018), less social support (World Health Organization, 2016) and poorer access to health services (Australian Institute of Health and Welfare, 2019). Thus, understanding how SES and geographical location influence the implementation and effectiveness of such interventions is crucial. Conducting process evaluations can be highly beneficial in this regard, as they aim to understand implementation and contextual factors that may act as facilitators and/or barriers to participation and influence intervention outcomes across different settings (Moore *et al*., 2015).

Although digital interventions should ideally be received similarly (i.e. in terms of perceived satisfaction and use) across different socioeconomic groups and geographical locations (e.g. metropolitan and regional areas), disadvantaged adolescents may not benefit as much from interventions as their more advantaged counterparts due to the aforementioned barriers. Thus, process evaluations are critical for determining whether interventions meet the needs of disadvantaged adolescents and for identifying challenges and areas requiring refinement to optimize intervention acceptability and efficacy (Ellard and Parsons, 2010). However, few process evaluations of digital interventions targeting lifestyle risk behaviours among disadvantaged adolescents have been conducted. For instance, in a recent systematic review of digital interventions targeting diet, alcohol and tobacco use among disadvantaged adolescents, only eight of the 14 studies included reported process evaluation outcomes (Egan *et al*., 2024a). Although most studies reported high participant satisfaction with the interventions, the feasibility, fidelity and acceptability of studies were mixed. Only five of the eight studies reported process evaluations of school-based digital interventions in the USA (*n* = 3), Spain (*n* = 1) and Australia (*n* = 1), with the remaining being community-, family- or clinic-based digital interventions. The Australian-based study was the only study to include process evaluation data from both students and teachers, providing insights on both how the intervention was experienced by the target user (students) and teachers who have a role in supporting the implementation of the intervention in the school-setting. However, data were collected and analysed between 2012 and 2014 and only among low SES boys in New South Wales (NSW), thus pointing at the need for more recent process evaluations that also include disadvantaged adolescents from various geographical locations across Australia. The dearth of process evaluations on this topic highlights a critical gap in understanding the implementation of digital interventions among disadvantaged adolescents.

In a recent process evaluation of *Health4Life*, a universal digital school-based program targeting multiple lifestyle risk behaviours such as poor diet, alcohol and tobacco use, high SES adolescents reported greater odds of using skills they learned from the intervention in the future compared to low SES adolescents (Champion *et al*., submitted for publication). This suggests that SES may significantly influence the uptake and utilization of intervention components. Although there were no significant differences in learned skills utilization between regional and metropolitan areas, a moderation analysis of *Health4Life* revealed that geographical location moderated the intervention’s effect (Egan *et al*., 2024b). Specifically, in regional areas, adolescents in *Health4Life* reported greater odds of reporting poor diet compared to the control. Although SES has not generally been found to moderate intervention effects on other school-based interventions targeting lifestyle behaviours such as diet (Yildirim *et al*., 2011) or physical activity (Robbins *et al*., 2020; Wassenaar *et al*., 2021). Therefore, additional investigation into the implementation outcomes among disadvantaged adolescents in *Health4Life* is warranted, particularly as these findings point at the complexity of intervention delivery across diverse contexts.

Against this backdrop, this study aims to conduct a comprehensive process evaluation of *Health4Life* among a sub-sample of low SES and regional adolescents across Australia, and their teachers, to elucidate the acceptability and feasibility of the intervention within these specific demographic contexts. Additionally, it seeks to identify areas for improvement and refinement, ultimately enhancing the intervention’s effectiveness and relevance for disadvantaged adolescents.

## METHODS

### Study design

The full study design and procedure of *Health4Life* are reported in the published protocol (Teesson *et al*., 2020). Briefly, the Health4Life Study is large cluster randomized controlled trial aimed at reducing chronic disease risk and improving physical and mental health among adolescents that was implemented in 71 secondary schools across Australia in 2019 (6639 participants aged 11–14 years at baseline) (Champion *et al*., 2023). Data were collected at baseline, post-intervention, 12, 24 and 36 months after baseline via self-report online or hardcopy questionnaires during class, with students and teachers in the *Health4Life* group completing process evaluation measurements at post-intervention. The current study examines a subset of post-intervention process evaluation questionnaire data from students and teachers from low SES and/or regional schools.

### Participant details

Seven intervention schools from low SES and/or regional areas were eligible for the current study. Low SES schools were identified based on their 2019 Index of Community Socio-educational Advantage (ICSEA) percentile score being in the bottom quartile (Australian Curriculum Assessment and Reporting Authority, 2023). ICSEA values, calculated by the Australian Curriculum, Assessment and Reporting Authority (ACARA), indicate a school’s relative educational advantage or disadvantage, based on factors such as parental occupation and education, the percentage of Aboriginal and/or Torres Strait Islander student enrolment, and student and school’s geographic remoteness. Schools with an ICSEA percentile < 25% were considered more educationally disadvantaged than at least 75% of schools in Australia. Regional school classification included schools outside of major cities according to the Australian Statistical Geography Standard Remoteness Structure, which categorizes regions based on their access to services (Australian Bureau of Statistics, 2021). Accessibility decreases as remoteness increases, with areas classified as *inner regional*, *outer regional*, *remote* or *very remote*.

As no student or teacher data were collected from one of the eligible schools, the final sub-sample comprised six intervention schools. Three of these schools (50%) fell under the category of inner or outer regional, two (33.3%) were classified as low SES only and one (16.7%) met criteria for both regional and low SES classifications. The majority of these schools were government (83.3%) with one being an independent school (16.7%). Five schools were located in NSW (83.3%), and one was in Western Australia (WA) (16.7%).

#### The Health4Life intervention

Schools allocated to the intervention group implemented *Health4Life* during regular health education classes. The program consists of six co-designed 20-min online modules, ideally delivered 1-week apart with students completing them individually (Champion *et al*., 2020). Underpinned by social influence, social cognitive and self-determination theories to promote healthy behaviour change, the core component of each module features interactive cartoon storylines addressing health risk behaviours for chronic disease including poor diet, alcohol use, tobacco smoking, physical inactivity, poor sleep and sedentary recreational screen time. The cartoon storylines interweave evidence-based information about health and social consequences of these behaviours, while also promoting resistance skills, normative education and autonomous motivation that underpin behavioural change theory (Michie *et al*., 2015). After completing each module, students complete short online quizzes to assess their knowledge. To reinforce content covered, factsheets for each module are available to both teachers and students and optional teacher-facilitated activities including online worksheets and homework tasks are provided. Students received personalized web-based feedback after each questionnaire on their adherence to national health guidelines. Additionally, students could access a companion smartphone app for further support and resources (Thornton *et al*., 2021). An overview of module content is provided in the Supplementary Appendices (p. 3).

### Process evaluation measures

#### Acceptability and areas for improvement

##### Student evaluation

Based on evaluation surveys from previous school-based prevention trials (Vogl *et al*., 2009; Newton *et al*., 2010; Teesson *et al*., 2017), students completed an online survey featuring eight questions assessing their satisfaction with *Health4Life*, content relatability and program utility. Example questions included, *‘Overall, how would you rate the Health4Life program?’* (‘Very good’, ‘Good’, ‘Average’, ‘Poor’, ‘Very poor’), *‘How relevant were the stories to experiences in your own life’* (‘Completely relevant’, ‘Somewhat relevant’, ‘Unsure’, ‘Somewhat irrelevant’, ‘Completely irrelevant’), *‘How likely are you to use the skills and information taught in the program in your own life’* (‘Very likely’, ‘Likely’, ‘Unsure’, ‘Unlikely’, ‘Very unlikely’). Responses were converted into binary variables to determine positive or unsure/negative evaluations. Two additional open-ended questions asked participants to provide feedback on one positive and one negative aspect of the program, capturing qualitative insights on the facilitators and barriers to program acceptability, respectively. The full student evaluation survey is provided in the Supplementary Appendices on pages 4 and 5.

##### Teacher evaluation

Teachers completed a 12-question online or hardcopy evaluation survey assessing program satisfaction and quality, ease of implementation and use, student satisfaction and engagement and likelihood of using and recommending *Health4Life*. Example questions included, *‘How would you rate the Health4Life program in comparison to other school-based health education programs?’* (‘Much better than most programs’, ‘Better than most programs’, ‘The same as most programs’, ‘Worse than most programs’, ‘A lot worse than most programs’), ‘*How easy did you find it to implement the Internet-based component of Health4Life program?’* (Very easy’, ‘Easy’, ‘Average’, ‘Difficult’, ‘Very difficult’), ‘*How much do you think the students liked the cartoon-based stories?’* (‘Liked a lot’, ‘Liked a little’, ‘Average’, ‘Disliked a little’, ‘Disliked a lot’), *‘How likely would you be to use the Health4Life course as a teaching resource in the future?’* (‘Very likely’, ‘Likely’, ‘Undecided’, ‘Unlikely’, ‘Very unlikely’). Two additional open-ended questions asked participants to provide feedback on improving the program could be improved and any additional comments about the modules. The full teacher evaluation survey can be found in the Supplementary Appendices on pages 6–9.

#### Fidelity and feasibility

Teachers were asked to complete an online or hardcopy version of a teacher logbook survey, to assess whether the six *Health4Life* modules and corresponding intervention components were implemented as intended (fidelity) and the practicality of implementing *Health4Life* (feasibility). The survey included a range of questions focused on several fidelity measures including adherence (i.e. whether students completed the online cartoon storylines for each module, and if teachers reviewed student summaries with their class or instructed students to download them), dose (i.e. the number and selection of activities students completed) and quality (i.e. teachers’ perceptions of the level of student engagement for each module). To ensure consistent delivery across all schools, the research team monitored intervention schools’ completion of modules via the intervention website and encouraged teachers to dedicate class time for their students to complete the module if it had not been completed in the scheduled week. Lastly, an open-ended question invited teachers to share any comments or concerns they had about each module. Further details about the questions asked in the teacher logbook survey can be found in the Supplementary Appendices on pages 10–16.

#### Analysis

Quantitative data were descriptively analysed in IBM SPSS Version 28.0.0.0 (190) to provide sample characteristics information and percentage agreement with quantitative survey item questions. Qualitative analysis of both student and teacher open-ended responses were conducted in Microsoft Excel and followed a codebook thematic analysis approach (Clarke and Braun, 2017; Braun and Clarke, 2021) to further describe the acceptability and feasibility of the intervention among disadvantaged adolescents. This approach was chosen to facilitate multiple team members coding different sections of the data efficiently. For student responses, the researchers immersed themselves in the data, repeatedly reviewing it to gain familiarity. Through an iterative and inductive approach, initial codes were generated and refined to create a codebook prior to discussing it with the authorship team. This codebook was then used to systematically code all open-text student survey responses to allow for a coherent and interpretive analysis of the data. The first author continued to develop themes, which were then reviewed, defined and named collaboratively with the authorship team. Similarly, for teacher responses, the first author coded each open-text response, adjusting codes as needed to describe developing insights of those data. Salient themes with explanatory value were then constructed, defined and named. In collaboration with the authorship team, themes were discussed and further refined resulting in a shared understanding of where the intervention fell short in meeting the needs of disadvantaged adolescents, along with suggestions for improvement.

## RESULTS

Of the 364 students attending the six schools in the current study, 214 (58.8%) completed the student evaluation surveys after completing the *Health4Life* program. The mean age of students was 12.77 (SD = 0.48), predominantly identifying as male (67.5%) and based in NSW (96.7%). Further details on student characteristics are reported in the Supplementary Appendices (p. 17). Regarding teacher participation, out of the 25 teachers across these six schools who registered an account on the *Health4Life* website, 14 (56%) completed both the teacher logbook and evaluation survey, two (8%) completed the teacher evaluation survey only and one (4%) completed the teacher logbook only.

### Student evaluation

Students’ overall response to *Health4Life* was generally positive, with 66.4% (142/214) of students rating *Health4Life* as good or very good. A total of 65.7% (140/213) found the information helpful, and 80.8% (172/213) reported that the skills and information they received in the program will help them to be healthy in the future. However, only 52.1% (111/213) found the stories relevant to their own experiences and 44.1% (94/213) reported that they would recommend *Health4Life* to their friends. Further details about student responses are in the Supplementary Appendices (p. 17).

Based on 213 student responses to ‘one good thing’ about *Health4Life*, we developed three dominant themes regarding facilitators for *Health4Life* acceptability (summarized in Table 1). Briefly, students commonly expressed appreciation for the educational value of *Health4Life*, citing informative evidence-based information on health concepts, and helpful and practical assistance for making healthy choices. Many found the stories and content engaging, with some describing it as relevant and relatable. Furthermore, students commended the engagement and interactivity of *Health4Life*, perceiving the cartoons conveyed information in an entertaining and accessible way, the program was fun, easy to use and the online format offered convenience and a different learning experience. However, four students reported ‘nothing good’ about *Health4Life* and 24 responses were indecipherable, suggesting they were potentially disengaged with the program or their response rate was influenced by procedural factors on how the evaluation survey was administered not facilitating quality responses (e.g. they may have been encouraged by their teacher to complete the survey at home if they were not present in class, which has been found to decrease response rates (Bidonde *et al*., 2023), or they may have had limited time to complete the survey in class due to unexpected circumstances, affecting response quality).

**Table 1:** Summary of dominant themes regarding facilitators for Health4Life acceptability from open-ended student responses

Themes and sub-themes	Description	Example quote
Educational value		
Informative (*n* = 48)	Informative evidence-based information on health-related concepts and the consequences of unhealthy behaviours	‘*I really like all the facts and statistics*’
Helpful (*n* = 41)	Helpful for making healthy choices	‘*it can help me in the future to stay healthy’.*
Stories and content		
Stories (*n* = 23)	Engaging stories**—**particularly backstory and character development	‘*It has a good story line*’
Relevant and relatable (*n* = 12)	Relevant and relatable to students’ lives and interests	‘*It was very relevant and it was something the class WANTED to do, not just another bit of school work*’.
Engagement and interactivity		
Cartoons (*n* = 19)	Cartoons conveyed information in an entertaining and accessible way	‘*the cartoons were pretty interesting i could’nt wait until i got into pdhpe so i could continue the story*’
Fun (*n* = 11)	Fun way to learn, preferable to traditional classwork	‘*it was fun but i leart alot*’
Easy (*n* = 7)	Easy to use and understand concepts taught	‘*It was easy to understand because it was a cartoon*’.
Online (*n* = 4)	Convenient online format without extensive written work	‘*use a computer’*

We developed four key themes regarding barriers to *Health4Life* acceptability based on the 214-student open-text responses to ‘one bad thing’ about *Health4Life* (summarized in Table 2). The overarching theme revolves around the need for improvement in content delivery and the technical execution of the program for this sub-sample of students. A prominent aspect relates to content quality, where some students highlighted issues related to relevance (in contrast to findings above), storyline coherence and perceived certain aspects as boring. Furthermore, there were structural concerns, particularly regarding the length of modules, relevance of questions and contradiction between the program messaging to reduce screen time and increase physical activity when completing the program online during Health and Physical Education lessons. Technical and design-related issues, including website functioning, graphic design and accessibility concerns were frequently reported. Some students expressed overall dissatisfaction with the entire program, while others mentioned the required engagement, such as homework tasks, as a source of discontent. However, 41 responses were indecipherable/nonsensical.

**Table 2:** Summary of key themes regarding barriers to Health4Life acceptability from open-ended student responses

Themes and sub-themes	Description	Example quote
Content quality		
Relevance/relatability (*n* = 16)	Lacked relevance, relatable language, diverse perspectives and real challenges students face	‘*some of the info doesn’t really include country kids*’
Boring (*n* = 15)	Boring aspects including repetitive, slow-paced, overall unengaging content	‘*it was boring sometimes*’
Storyline issues (*n* = 14)	Storyline issues including confusing, unrealistic scenarios, lacked long-term character outcomes, potentially encouraged unhealthy behaviours	‘*that its cringy*’
Structural concerns		
Too long (*n* = 20)	Modules were too long	‘*the modules went for too long i believe there should only be 3-4 modules’.*
Too short (*n* = 11)	Modules were too short, needed more detailed storylines and character development	‘*it should go for longer*’
Contradiction between program format and health-promoting messaging (*n* = 5)	Contradiction between *Health4Life* promoting physical activity and reduced screen time when using screens for program	‘*you are telling us to be more active yet we spend hours on a screen completing the activities*’
Technical and design issues		
Website functioning (*n* = 13)	Website functioning issues including slow loading times, glitches and lag	‘*The lag and glitches of the website*’
Graphic design (*n* = 11)	Unappealing graphic design including character style, and text outside speech bubble borders	‘*Bad graphics*’
Accessibility (*n* = 8)	Accessibility impacted by too much text, lack of voice overs and reliance on internet access	‘*not all people can read or have internet*’
Overall dissatisfaction		
Everything (*n* = 5)	Everything is unsatisfactory	‘*all of it*’
Required engagement (*n* = 3)	Required engagement with materials including homework participation	‘*being made to do it as homework*’

### Teacher evaluation

Most of the 16 teachers who completed the teacher evaluation survey rated *Health4Life* positively (14/16; 87.5%) and found the internet-based component easy to implement (14/16; 87.5%). A total of 81.3% (13/16) perceived the cartoon stories held the students’ attention well/very well, and considered the educational quality of the additional classroom activities in the online teacher centre to be good to very good (14/16; 87.5%). However, only half would recommend *Health4Life* to others (8/16; 50.0%). Further details of teacher survey responses to quantitative questions are in the Supplementary Appendices (p. 18). In response to how *Health4Life* could be improved for the future some teachers perceived the program was good as is, stating ‘*I don’t think there is anything at this stage that I could suggest, it is a great program’*, whereas others provided suggestions regarding accessibility, the user interface and experience, engagement and relevance, timeframe expectations for users and content clarity and appropriateness.

#### User interface and experience (n = 10)

Similar to students, teachers suggested addressing website glitches as this impacted user experience, and ensuring speech bubble text on cartoon slides fits within borders. They recommended removing the repetitiveness of student worksheets, making them dynamic and embedding them within the *Health4Life* platform to eliminate the need for printing. Moreover, teachers proposed adding minimum time requirements for each cartoon slide to assist with getting students to work at similar paces and prevent them skipping ahead without engaging with the content. Although teachers noted that questions and interactive activities embedded within the modules helped engage students.

*Found it hard to stop some students from skipping through the cartoon without reading it properly. The use of questions and interactive activities through the online modules engaged the students*. (Teacher)

Teachers also suggested streamlining administrative tasks by increasing teacher account privileges for resetting student passwords quickly and efficiently and the ability to set generic passwords for individual classes.

#### Engagement and relevance (n = 5)

Teachers praised the engaging stories that seamlessly integrated essential information, whilst maintaining student interest across different ability levels. However, some expressed that despite *Health4Life* providing students with potential benefits, student apathy towards new learning experiences is common, indicating a need for a more engaging program. A few teachers discussed variations in student engagement and module completion, with students either racing through independently or struggling to finish them.

*The engagement and completion of modules depended on the ability of students. Some students raced through and even completed the modules at home (even though they had been told to complete them in class) while other students did not complete all modules….* (Teacher)

#### Content clarity and appropriateness (n = 5)

Teachers suggested improving content clarity and assessing the appropriateness of some content. Specifically, concerns were raised regarding the difficulty in understanding some of the questionnaires, particularly the sleep questionnaire. Additional feedback included perceiving some activities as overly simplistic and requiring further information to improve their educational value, more content or longer lessons to cover one school term and redistributing the workload across the modules as some had too much content and activities whereas others were lacking. Therefore, a thorough review and testing of all materials are needed to ensure they address these concerns effectively.

*We found some modules had too much content/activities and others didn’t have enough for a whole lesson. Perhaps adjusting the amount of work in some lessons would improve …*. (Teacher)

#### Accessibility (n = 4)

Some teachers noted limited access to computer labs in certain schools, which could impact participation for students without personal devices. Others commented on the challenge of balancing using the program as prescribed with catering to individual student learning needs. Moreover, they discussed the importance of voiceovers to accompany the cartoon slides, not only to support students with lower literacy levels but also to improve engagement. Therefore, an offline or adapted format may be necessary to overcome these issues to ensure *Health4Life* is accessible to students with diverse backgrounds and learning needs.

*Audio options for students who have poor reading skills.* (Teacher)

*… Easy to access and use. My school each high school student has to have a device so makes access easy- in other schools where this is not the case it may have proved harder to access computer labs*. (Teacher)

#### Timeframe expectations for users (n = 2)

Lastly, teachers mentioned discrepancies between advised and actual module completion timeframes, indicating more accurate instruction is required to aid in lesson planning. Some teachers facing time constraints chose to double up lessons to cover the curriculum. To ensure more teachers understand the realistic timeframes and program format flexibility, and accommodate varying time constraints, clear documentation of this must be included in the program implementation guide available to teachers.

*… we were told around 20 minutes per module- realistically it took around 40 minutes for our Year 7 students to read each module. Just advising of a more realistic timeframe*. (Teacher)

### Teacher fidelity

Teacher logbook data collected from 16 teachers are reported in Supplementary Appendix Table 6 (p. 19). Most teachers indicated that the cartoon storylines were fully completed, except for modules 5 and 6, primarily due to lack of time. Teachers reported students were somewhat to very engaged 85.7–100% across modules, with the greatest engagement reported for module 3 (100%). Module 3 also had the most reviewed summary sheets for students and teachers (93.3%). Module 4 demonstrated the highest completion rates for both the online interactive activity (86.7%) and the three offline activities (88.9%). None of the recommended/homework tasks across modules were fully completed. Teachers expressed concerns about accessibility, login issues and too much content in module 1, while module 2 feedback pertained to graphic design issues affecting text readability and the need for more content. Modules 5 and 6 were positively reviewed, with module 5 complimented for its engaging activities, particularly the interactive sleep hazards activity, while module 6 was commended as a ‘*good summative lesson*’ (Teacher).

## DISCUSSION

Few studies have reported on process evaluations of school-based digital interventions targeting poor diet, alcohol use and tobacco smoking among disadvantaged adolescents (Egan *et al*., 2024a), leaving a significant gap in the literature regarding the implementation and relevance of such interventions for this population. The current study reports on the implementation of a universal school-based digital program targeting multiple behaviours among disadvantaged adolescents in Australia. The *Health4Life* intervention received positive feedback from both teachers and students, yet only half of teachers and 44.1% of students expressed intentions to recommend the program to others. This is in contrast to the process evaluation among the entire sample which demonstrated a more positive overall experience and higher rates of recommendation (Champion *et al*., submitted for publication). This highlights the need to adapt and optimize interventions with the distinct population in mind. Several areas for improvement and refinement were identified in the present study to improve the effectiveness and relevance of the intervention for disadvantaged adolescents.

Compared to process evaluation findings from the entire sample, this sub-sample of students evaluated *Health4Life* less favourably. For instance, a smaller proportion of students rated the program positively (66% versus 75% among the entire sample), enjoyed the stories (63% versus 75%), found the information helpful (65% versus 76%), were likely to use the skills and information in their own life (60% versus 70%), and would recommend *Health4Life* to friends (44% versus 50%). Despite the sub-sample of students in the current study reporting that the entertaining online cartoons made health messaging accessible, these lower ratings suggest the program has limited relevance for disadvantaged adolescents. Interestingly teacher evaluations of *Health4Life* in this study aligned closely with those of the entire sample and were mostly positive. Teacher fidelity data were also comparable to the entire sample, with similar levels of student engagement (~92% versus ~85%) and slightly more activities completed per module (3 versus 2). However, only 50% of these teachers would recommend *Health4Life* to others compared to 64% in the entire sample. The discrepancy may reflect teachers’ recognition of *Health4Life*’s value and potential in low SES or regional contexts where comparable programs may be scarce, whilst acknowledging the need for content and technical delivery improvements which was also noted in the entire sample process evaluation, alongside *Health4Life*’s limited contextual relevance for disadvantaged adolescents. These findings are consistent with other school-based digital interventions for disadvantaged adolescents. A recent systematic review reported that while participants generally expressed high satisfaction with the interventions, the feasibility, fidelity and acceptability of studies were mixed (Egan *et al*., 2024a). For instance, in one study (Smith *et al*., 2014; Lubans *et al*., 2016) only 44% agreed or strongly agreed they enjoyed using the intervention app, potentially due to limited engaging features and lack of co-design. Another study (Nollen *et al*., 2014) reported high enjoyment scores (mean = 4.5/5; SD = 0.9) and participant engagement with the program on 63% of days, which may be due to conducting in-depth co-design with the target population and addressing their needs. It is worth noting that the teacher-reported fidelity data regarding students’ *Health4Life* module completion (i.e. 85.7–100% completed module cartoon storylines) is similar to intervention completion of other school-based digital programs among disadvantaged adolescents (Egan *et al*., 2024a). These completion rates coupled with high teacher-reported rates of student engagement (~92%) are positive particularly as engagement is a key element for improved intervention effects, albeit only 64% (16/25) of teachers completed the teacher logbook.

Themes developed from the qualitative data in the current study revealed key issues resonating with challenges faced by low SES/regional adolescents. For instance, students critiqued *Health4Life’*s content quality and relevance of stories to their own experiences. They raised structural concerns about the program’s length and the requirement to engage with *Health4Life* via computers during health education classes, which seemed discordant with its healthy behaviour messaging. Both students and teachers criticized the accessibility of the program for diverse backgrounds and literacy levels, echoing broader challenges low SES and regional adolescents face. Differences in digital literacy among adolescents from different socioeconomic backgrounds, including how they access and use technology, may have shaped how relevant the intervention was to them. Indeed, Lim *et al*. (2024) have suggested that low SES adolescents engage with digital media differently compared to their more advantaged peers, which may influence their digital literacy. For example, in one study (Harris *et al*., 2017) there were differences in computer use among a sample of adolescents in Western Australia. Specifically, low SES adolescents were less likely to access computers in school than high SES adolescents. Additionally, they were more likely to use their home computer for non-academic activities such as chat rooms and multimedia (e.g. video and music). Whereas high SES adolescents used their home computer learning programs to improve their academic skills. Considering this, incorporating support mechanisms such as greater visual material and chatbots may be potentially helpful features to compensate for SES differences (Maenhout *et al*., 2022).

Nevertheless, this study identified refinements needed to improve the student and teacher experience, including: adding dynamic features (e.g. embedding activity worksheets); more appropriate content (e.g. addressing repetitive worksheets); clearer documentation of program flexibility to accommodate time constraints; improving accessibility (e.g. voiceovers, reducing text on screen); fixing issues such as website glitches and text overflowing borders; setting minimum time requirements on cartoon slides to ensure students work at similar paces and granting teachers greater administrative privileges (e.g. reset passwords easily). For improved implementation suggested refinements include: balancing the workload across modules (e.g. content redistribution and additional materials for certain modules), catering better to diverse learning needs and environments (e.g. offering flexible delivery modes with more offline content) and providing resources to improve digital literacy (e.g. such as step-by-step instructions on how to log in and use the website—both written and via a recorded video—, adding a chatbot to allow for instant assistance). These points suggest that without adapting *Health4Life* to disadvantaged adolescents’ context, their sustained engagement and the program’s utility may be compromised.

Indeed, adapting *Health4Life* to the specific skills, needs and barriers of disadvantaged adolescents is essential for ensuring its relevance and for promoting effective engagement with the intervention (Yardley *et al*., 2016). This requires contextual adaptation leveraging co-design approaches, including disadvantaged adolescents and their teachers as support, to appropriately address feedback and refine the program content, delivery methods and engagement strategies. Involving both students and their teachers in the adaptation process is important as they may experience the program differently. For instance, the program’s design and format may align more closely with teachers’ pedagogical perspectives on targeting lifestyle risk behaviours among adolescents than with the students’ real-life experiences. Nonetheless, considering that teachers facilitate the implementation of *Health4Life* with their students, ensuring their experience is seamless and enjoyable is paramount to its success.

Although *Health4Life* was co-designed with students and teachers, recruitment for this development phase lacked a representative mix of schools with varying SES and geographic distribution, predominantly involving metropolitan and more affluent schools. This may partly explain the less favourable process evaluation outcomes in the current study. Nevertheless, moving forward, the co-design adaptation process with disadvantaged adolescents and their teachers should be iterative with development and implementation intertwined to allow for ongoing refinement and facilitate participation. Al-Dhahir *et al*. (2022) identified this approach as a facilitator in digital lifestyle intervention participation among low SES adults, and it could have addressed early issues with *Health4Life* teachers in the current study mentioned, including student difficulties with logging in and registering. Confidentiality and privacy concerns are also important factors to consider. They have been identified as prerequisites for participation in digital mental health programs among vulnerable young people, specifically school leavers, which is associated with experiencing socioeconomic disadvantage (Kuosmanen *et al*., 2018). Furthermore, incorporating the broader social networks of disadvantaged adolescents, including parents and peers may improve the acceptability and feasibility of the intervention. This is because peers can influence adolescent behaviours and peer-led interventions can be effective at targeting lifestyle risk behaviours (Veenstra and Laninga-Wijnen, 2022). Similarly, parental factors and behaviour modelling can influence adolescent behaviours (e.g. dietary composition) and parent-based interventions delivered alongside school-based programs have shown success in improving adolescent outcomes (Champion *et al*., 2022; Osman *et al*., 2024). The *Health4Life* team are currently evaluating the accompaniment of a parent-based program with *Health4Life* among disadvantaged adolescents.

### Strengths and limitations

This study has several strengths, including gathering detailed feedback from both disadvantaged students and their teachers to provide an understanding of *Health4Life*’s acceptability for this demographic and areas for refinement. The inclusion of schools from diverse regions, including resource-constrained settings, enhances the relevance of the findings across various educational contexts. The mixed-methods approach, combining quantitative data (e.g. program ratings) with qualitative feedback enriched the analysis to provide deeper insights into how and why *Health4Life*’s acceptability and implementation differed for disadvantaged adolescents. These findings have important implications for designing and delivering interventions to different subgroups, including low SES and regional adolescents.

However, there are some limitations. Teachers from one inner regional independent WA school did not complete the teacher logbook nor the teacher evaluation survey, limiting comparative analysis with the one outer regional WA school that provided data. The WA teachers who provided data only completed the teacher evaluation and did not submit any teacher logbook data, restricting our understanding of *Health4Life* in this region. The use of teacher-led surveys may have introduced response bias, with potentially more engaged and motivated teachers actively providing evaluation and logbook data. Thus, future studies should triangulate data with objective measures (e.g. from the intervention website) to corroborate teacher survey responses and provide information for teachers who do not complete the surveys. Only one author coded qualitative teacher data which may have limited richness of analysis, although thematic analysis does not always require multiple reviewers (Byrne, 2022). Finally, the findings may not be fully generalisable due to the limited number of schools and regions involved in the study. Future research with a broader sample is needed to validate these results.

## CONCLUSION

This study addresses a notable gap in the literature by examining the implementation and relevance of a universal school-based digital intervention targeting lifestyle risk behaviours among disadvantaged adolescents. The findings reveal key areas for refinement including improving content relevance and technical execution to better resonate with disadvantaged adolescents, and ensuring accessibility for students with diverse backgrounds and learning needs. Future iterations of *Health4Life* should leverage iterative co-design approaches involving both disadvantaged adolescents and teachers to refine the program and ultimately enhance its engagement, relevance and effectiveness. The implications of the study findings have broader applicability in providing valuable guidance for optimizing the design and implementation of digital interventions among diverse populations.

## Supplementary Material

daae170_suppl_Supplementary_Appendices

## Data Availability

The data underlying this article cannot be shared publicly for the privacy of individuals who participated in the study.
