# Supplementary material for: Process evaluation of the digital Health4Life intervention among a sample of disadvantaged adolescents and teachers
Source: Health Promot Int. 2024 Nov 29;39(6):daae170. doi: 10.1093/heapro/daae170 (PMC11606168; doi:10.1093/heapro/daae170)
Supplement: daae170_suppl_Supplementary_Appendices [file daae170_suppl_supplementary_appendices.docx]

**Supplementary materials**

**Table of contents**

[Appendix Table 1: School Characteristics 2](#_Toc172550371)

[Overview of *Health4Life* module content 3](#_Toc172550372)

[Student evaluation survey 4](#_Toc172550373)

[Teacher evaluation survey 6](#_Toc172550374)

[Teacher logbook survey (implementation data) 10](#_Toc172550375)

[Appendix Table 2: Student characteristics 17](#_Toc172550376)

[Appendix Table 3: Student responses to quantitative evaluation survey questions 18](#_Toc172550377)

[Appendix Table 4: Teacher responses to quantitative evaluation survey questions 19](#_Toc172550378)

[Appendix Table 5: Summary of engagement with Health4Life components based on teacher logbook responses. 20](#_Toc172550379)

# Appendix Table 1: School Characteristics

| Characteristic |  |
| --- | --- |
| State (N(%)) |  |
| New South Wales | 5 (83.3%) |
| Western Australia | 1 (16.7%) |
| School ICSEA quartile (N(%)) |  |
| First | 3 (50.0%) |
| Second-Fourth | 3 (50.0%) |
| Geographic remoteness |  |
| Inner or outer regional | 4 (66.7%) |
| Major city | 2 (33.3%) |
| School sector |  |
| Government | 5 (83.3%) |
| Independent | 1 (16.7%) |

# Overview of *Health4Life* module content

Appendix Table 3

| Lesson | Content |
| --- | --- |
| 1 | - Guidelines for eating healthily and benefits of a healthy diet. - Sleep needs for adolescents and benefits of sleeping well. - Guidelines for recreational screen time and benefits of limiting screen use. |
| 2 | - Reasons why young people choose to, or not to, drink alcohol/smoke. - Reducing harms from alcohol use and smoking. - Strategies to resist peer pressure. - Benefits of physical activity. |
| 3 | - Short and long-term consequences of alcohol and tobacco use. - Consequences of excessive sedentary recreational screen time. - Strategies to reduce sedentary recreational screen time. - Responsible use of social media. |
| 4 | - Social, financial and legal consequences of alcohol and tobacco use. - Assertive communication and refusal skills. - Guidelines for physical activity and sedentary behaviour. - SMART goal setting. |
| 5 | - Understanding food labels and serving sizes. - Limiting sugar-sweetened beverage consumption. - Improving sleep hygiene. - Benefits of sleeping well and prioritising sleep when needed. |
| 6 | - Associations and interrelations between health habits. - Relationships between the Big 6 and mental health. - Physical, social and emotional benefits of health and wellbeing. - The ‘Big 6’ and long-term health. |

# Student evaluation survey

**
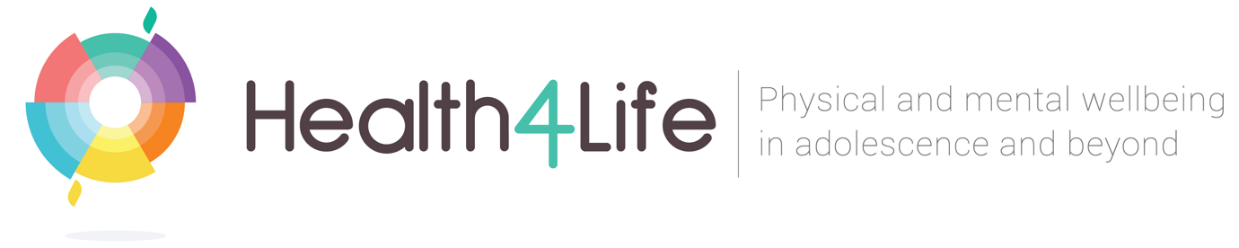
**

**Health4Life Program – Online Student Evaluation**

Thank you for completing the *Health4Life program*. We would now like to get your feedback about the program. Please answer the questions below.

***The questions below refer to the online cartoon lessons***

1. **Overall, how would you rate the Health4Life program?**

Very Good

Good

Average

Poor

Very Poor

1. **How much did you like learning in this way (i.e. via online cartoon lessons)?**

Liked a lot

Liked a little

Neither liked nor disliked

Disliked a little

Disliked a lot

1. **How much did you like the stories in the Health4Life lessons?**

Liked a lot

Liked a little

Neither liked nor disliked

Disliked a little

Disliked a lot

1. **How relevant were the stories to experiences in your own life?**

Completely relevant

Somewhat relevant

Unsure

Somewhat irrelevant

Completely irrelevant

1. **How helpful was the information to you?**

Extremely helpful

Somewhat helpful

Neither helpful or unhelpful

Somewhat unhelpful

Extremely unhelpful

1. **Do you think the skills and information you received in the program will help you to be healthy in the future?**

Yes, I think they will help a great deal

Yes, I think they will help somewhat

No, I don’t think they will help at all

I’m not sure whether they will help or not

1. **How likely are you to use the skills and information taught in the program in your own life?**

Very Likely Likely Unsure Unlikely Very Unlikely

1. **Would you recommend the Health4Life program to your friends?**

Yes No Maybe

1. **Please list ONE good thing about the Health4Life program:**

1. **Please list ONE bad thing about the Health4Life program:**

# Teacher evaluation survey

**
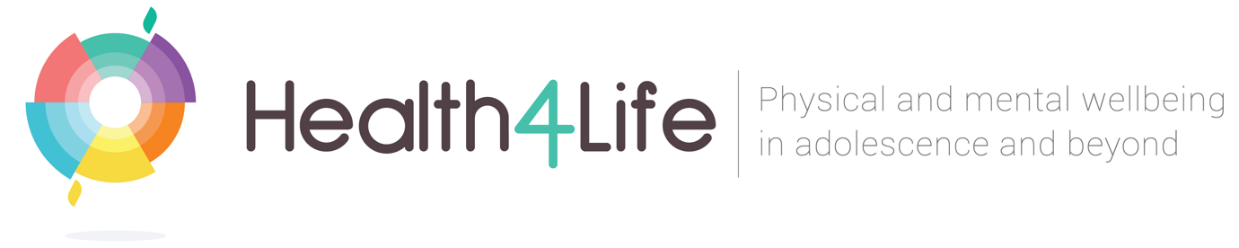
**

**Health4Life Program - Teacher Evaluation**

Thank you for completing the *Health4Life program*. We would now like to get your feedback about the program. Please answer the questions below.

1. **Overall, how would you rate the Health4Life program?**

Very good

Good

Average

Poor

Very poor

1. **How would you rate the Health4Life program in comparison to other school-based health education programs?**

Much better than most programs

Better than most programs

The same as most programs

Worse than most programs.

A lot worse than most programs

1. **How easy did you find it to implement the Internet-based component of Health4Life program?**

Very easy

Easy

Average

Difficult

Very difficult

1. **How easy was it for you to gain access to the computer facilities at your school for the Health and Physical Education classes?**

Very easy

Easy

Average

Difficult

Very difficult

1. **How well do you think the cartoon stories held the students’ attention?**

Very well

Well

Average

Poorly

Very poorly

1. **How well do you think the students could recall the health information after the cartoon-based stories?**

Very well

Well

Average

Poorly

Very poorly

1. **How much do you think the students liked the cartoon-based stories?**

Liked a lot

Liked a little

Average

Disliked a little

Disliked a lot

1. **How would you rate the educational quality of the additional classroom activities included in the online Teacher Centre?**

Very good

Good

Average

Poor

Very poor

1. **How easy did you find it to use the online Teacher Centre to prepare the activities for your class lessons?**

Very easy

Easy

Average

Difficult

Very difficult

1. **How well do you believe the additional classroom activities helped to reinforce the health information to students?**

Very well

Well

Average

Poorly

Very poorly

1. **How likely would you be to use the Health4Life course as a teaching resource in the future?**

Very likely

Likely

Undecided

Unlikely

Very unlikely

1. **How likely is it that you would recommend the Health4Life course to others?**

Very likely

Likely

Undecided

Unlikely

Very unlikely

1. **Could you please list any ways in which you think the Health4Life program could be improved for the future?**

1. **Please list any additional comments you have regarding the Health4Life modules**

# Teacher logbook survey (implementation data)

**study**

National Drug and Alcohol

Research Centre,

University of New South Wales

**
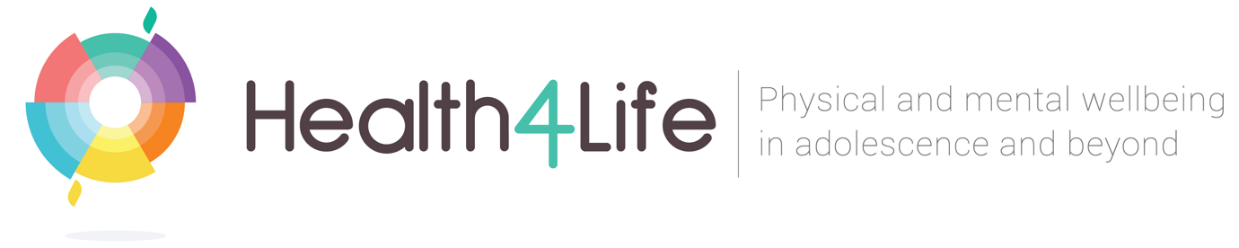
**

TEACHER LOG BOOK

**Teacher Name: School:**

**Please complete this logbook at the end of each *Health4Life* lesson.**

**When you have finished all six modules please return the completed logbook to**  Bridie Osman or Karrah McCann
via email ([Bridie.osman@sydney.edu.au](mailto:Bridie.osman@sydney.edu.au)) ([Karrah.mccann@sydney.edu.au](mailto:Karrah.mccann@sydney.edu.au)) post (Bridie Osman/ Karrah McCann, The Matilda Centre, Level 6, Jane Foss Russell Building, University of Sydney, Sydney NSW 2006, Australia).
 **Thank you!**

| MODULE 1 | Yes | No |
| --- | --- | --- |
| Did your students complete the online *Health4Life* cartoon component for Module 1? |  |  |
| Did your students complete the *whole* online cartoon for Module 1?  *If not, please give reasons why:* |  |  |
| Did you go through the Student Summary with your class for Module 1, or instruct them to download it? |  |  |
| On average, how would you rate the level of engagement of your students with this module?   - Very engaged - Somewhat engaged - Neither engaged nor disengaged - Somewhat disengaged - Very disengaged | | |
| Which activities did your students complete?   - What’s in the fridge: online activity - The Health4Life app download (homework task, recommended) - Stop and Think: Healthy food choices - Question time, screen time - Sleep and me | | |
| Did you prompt students to download the Health4Life app during class time? *If not, please give reasons why (e.g. school policy about mobile phones, lack of time):* |  |  |
| Did you encourage or remind students to use the Health4Life app outside of class time? *If so, how?* |  |  |
| Do you have any comments / concerns about Module 1? (*Please explain)* |  |  |

| MODULE 2 | Yes | No |
| --- | --- | --- |
| Did your students complete the online *Health4Life* cartoon component for Module 2? |  |  |
| Did your students complete the *whole* online cartoon for Module 2?  *If not, please give reasons why:* |  |  |
| Did you go through the Student Summary with your class for Module 2, or instruct them to download it? |  |  |
| On average, how would you rate the level of engagement of your students with this module?   - Very engaged - Somewhat engaged - Neither engaged nor disengaged - Somewhat disengaged - Very disengaged | | |
| Which activities did your students complete?   - What physical activities do you like? - Alcohol and smoking quiz - Alcohol, smoking and young people - Step counter | | |
| Did you prompt students to download the Health4Life app during class time? *If not, please give reasons why (e.g. school policy about mobile phones, lack of time):* |  |  |
| Did you encourage or remind students to use the Health4Life app outside of class time? *If so, how?* |  |  |
| Do you have any comments / concerns about Module 2? |  |  |

| MODULE 3 | Yes | No |
| --- | --- | --- |
| Did your students complete the online *Health4Life* cartoon component for Module 3? |  |  |
| Did your students complete the *whole* online cartoon for Module 3?  *If not, please give reasons why:* |  |  |
| Did you go through the Student Summary with your class for Module 3, or instruct them to download it? |  |  |
| On average, how would you rate the level of engagement of your students with this module?   - Very engaged - Somewhat engaged - Neither engaged nor disengaged - Somewhat disengaged - Very disengaged | | |
| Which activities did your students complete?   - Interactive quiz on teen drinking and smoking - Homework: My health journey so far (recommended) - Myths and facts about alcohol and tobacco - Using social media responsibly | | |
| Did you prompt students to download the Health4Life app during class time? *If not, please give reasons why (e.g. school policy about mobile phones, lack of time):* |  |  |
| Did you encourage or remind students to use the Health4Life app outside of class time? *If so, how?* |  |  |
| Do you have any comments / concerns about Module 3? |  |  |
| MODULE 4 | **Yes** | **No** |
| Did your students complete the online *Health4Life* cartoon component for Module 4? |  |  |
| Did your students complete the *whole* online cartoon for Module 4?  *If not, please give reasons why:* |  |  |
| Did you go through the Student Summary with your class for Module 4, or instruct them to download it? |  |  |
| On average, how would you rate the level of engagement of your students with this module?   - Very engaged - Somewhat engaged - Neither engaged nor disengaged - Somewhat disengaged - Very disengaged | | |
| Which activities did your students complete?   - Activity all around you - SMART goals - Sugary drinks quiz - Physical Activity researcher (homework) | | |
| Did you prompt students to download the Health4Life app during class time? *If not, please give reasons why (e.g. school policy about mobile phones, lack of time):* |  |  |
| Did you encourage or remind students to use the Health4Life app outside of class time? *If so, how?* |  |  |
| Do you have any comments / concerns about Module 4? |  |  |

| MODULE 5 | Yes | No |
| --- | --- | --- |
| Did your students complete the online *Health4Life* cartoon component for Module 5? |  |  |
| Did your students complete the *whole* online cartoon for Module 5?  *If not, please give reasons why:* |  |  |
| Did you go through the Student Summary with your class for Module 5, or instruct them to download it? |  |  |
| On average, how would you rate the level of engagement of your students with this module?   - Very engaged - Somewhat engaged - Neither engaged nor disengaged - Somewhat disengaged - Very disengaged | | |
| Which activities did your students complete?   - Healthy, happy zzzs - Sleep plan - What’s in my food? - Keeping track (homework) | | |
| Did you prompt students to download the Health4Life app during class time? *If not, please give reasons why (e.g. school policy about mobile phones, lack of time):* |  |  |
| Did you encourage or remind students to use the Health4Life app outside of class time? *If so, how?* |  |  |
| Do you have any comments / concerns about Module 5? |  |  |

| MODULE 6 | Yes | | No |
| --- | --- | --- | --- |
| Did your students complete the online *Health4Life* cartoon component for Module 6? |  | |  |
| Did your students complete the *whole* online cartoon for Module 6?  *If not, please give reasons why:* |  | |  |
| Did you go through the Student Summary with your class for Module 6, or instruct them to download it? |  | |  |
| On average, how would you rate the level of engagement of your students with this module?   - Very engaged - Somewhat engaged - Neither engaged nor disengaged - Somewhat disengaged - Very disengaged | | | |
| Which activities did your students complete?   - The Big link - A Healthy 24 hours - Help Seeker - Exercise your mood | | | |
| Did you prompt students to download the Health4Life app during class time? *If not, please give reasons why (e.g. school policy about mobile phones, lack of time):* | |  |  |
| Did you encourage or remind students to use the Health4Life app outside of class time? *If so, how?* | |  |  |
| Do you have any comments / concerns about Module 6? | |  |  |

# Appendix Table 2: Student characteristics

| **Characteristic** |  |
| --- | --- |
|  | **Total (n = 214)** |
| **Age (mean, SD)** | 12.77 (0.48) |
| **Gender n (%)** |  |
| Male | 143 (67.5%) |
| Female | 66 (31.1%) |
| Preferred not to disclose | 3 (1.4%) |
| **State n (% of participants)** |  |
| New South Wales | 207 (96.7%) |
| Western Australia | 7 (3.3%) |
| **School year group** |  |
| Year 7 | 210 (99.1%) |
| Year 8 | 2 (0.9%) |
| **Country born in** |  |
| Australia | 199 (93.9%) |
| Other | 13 (6.1%) |
| **Main language at home** |  |
| English | 210 (99.1%) |
| Other | 2 (0.9%) |
| **Grades – usually get in school** |  |
| 90-100% | 36 (17.0%) |
| 80-89% | 63 (29.7%) |
| 70-79% | 58 (27.4%) |
| 60-69% | 34 (16.0%) |
| 50-59% | 11 (5.2%) |
| 49% and below | 10 (4.7%) |

# Appendix Table 3: Student responses to quantitative evaluation survey questions

| **Student responses to quantitative survey questions** | **Number agreeing (%)** |
| --- | --- |
| Rated Health4Life as good or very good | 142/214 (66.4%) |
| Like learning this way (a little to a lot) | 137/213 (64.3%) |
| Like stories in Health4Life cartoons (a little to a lot) | 135/213 (63.4%) |
| Stories were relevant to own experiences (somewhat to completely) | 111/213 (52.1%) |
| Information was helpful (somewhat to extremely) | 140/213 (65.7%) |
| Skills and information will help them in future to be healthy (yes; somewhat to a great deal) | 172/213 (80.8%) |
| Likely to use the skills and information in own life (likely to very likely) | 129/213 (60.6%) |
| Would recommend Health4Life to friends (yes) | 94/213 (44.1%) |

# Appendix Table 4: Teacher responses to quantitative evaluation survey questions

| **Teacher evaluation survey responses** | **Number agreeing (%)** |
| --- | --- |
| Overall rating of Health4Life (good or very good) | 14/16 (87.5%) |
| Rating of Health4Life compared to other school-based health education programs (better than most) | 11/16 (68.8%) |
| Ease of implementing Health4Life internet-based component (easy to very easy) | 14/16 (87.5%) |
| Ease of access to computer for Health and Physical Education classes (easy to very easy) | 11/16 (68.8%) |
| Ability of cartoon stories to hold students’ attention (well to very well) | 13/16 (81.3%) |
| Students’ ability to recall health information after cartoon-based stories (well to very well) | 12/16 (75.0%) |
| Perceived students’ liking of cartoon-based stories (liked a little to a lot) | 12/16 (75.0%) |
| Educational quality of additional classroom activities in the online Teacher Centre (good to very good) | 14/16 (87.5%) |
| Ease of use of online Teacher Centre to prepare activities for class lessons (easy to very easy) | 15/16 (93.8%) |
| How well additional classroom activities helped to reinforce the health information to students (well to very well) | 12/16 (75.0%) |
| Likelihood of using Health4Life as a teacher resource in the future (likely to very likely) | 9/16 (56.3%) |
| Likelihood of recommending Health4Life to others (likely to very likely) | 8/16 (50.0%) |

# **Appendix Table 5:** Summary of engagement with Health4Life components based on teacher logbook responses.

| **Component** | **Description** | **Delivery method/Format** | **Completion/reach** |
| --- | --- | --- | --- |
| Cartoon storylines | 20-minute cartoon storylines that teach key prevention messages. | Online via *Health4Life* website | **Module 1**: 16/16 (100%); *engaged 93.8%  **Module 2**: 16/16 (100%); *engaged 93.8%  **Module 3**: 15/15 (100%); *engaged 100%  **Module 4**: 15/15 (100%); *engaged 86.7%  **Module 5**: 14/15 (93.3%); *engaged 93.3%  **Module 6**: 12/14 (85.7%); *engaged 85.7% |
| Summary sheets | Evidence-based summary sheets for students and teachers. | Online PDF | **Module 1**: 11/16 (68.8%)  **Module 2**: 11/16 (68.8%)  **Module 3**: 14/15 (93.3%)  **Module 4**: 12/15 (80%)  **Module 5**: 13/15 (86.7%)  **Module 6**: 10/14 (71.4%) |
| Activities (offline) | Activities to promote self-management and interpersonal skills. In modules 1-4, a specific activity is marked as the recommended task. | Offline; mixture of paired interviews, group work, class discussion, and worksheet format. | **Module 1**: (50%-87.5%)  **Module 2**: (25%-100%)  **Module 3**: (20%-100%)  **Module 4**: (80%-100%)  **Module 5**: (20%-100%)  **Module 6**: (64.3%-100%) |
| Activities (online) | One interactive activity per module to reinforce learning from cartoon. | Online interactive activity e.g. game, quiz, interactive worksheet | **Module 1**: *What’s in the Fridge?* (13/16; 81.3%)  **Module 2**: *What physical activities do you like?* (recommended) (13/16; 81.3%)  **Module 3**: *Interactive quiz on teen drinking and smoking* (13/15; 86.7%)  **Module 4**: *Activity All Around You* (13/15; 86.7%)  **Module 5**: *Healthy, happy zzz’s* (10/15; 66.7%)  **Module 6**: *The Big Link* (9/14; 64.3%) |

**Engagement percentages reflecting the proportion of students who were “somewhat to very engaged” with the component.*
